# Rural-urban differences in individual and environmental correlates of physical activity in Canadian adults

**DOI:** 10.1016/j.pmedr.2022.102061

**Published:** 2022-11-21

**Authors:** Chelsea Pelletier, Nicole White, Annie Duchesne, Larine Sluggett

**Affiliations:** a School of Health Sciences, University of Northern British Columbia, Prince George, British Columbia, Canada; b Department of Psychology, University of Northern British Columbia, Prince George, British Columbia, Canada; c University of Northern British Columbia, Prince George, British Columbia, Canada

**Keywords:** Rural-urban disparities, Exercise, Social-ecological model, Socioecological model, Barriers, Physical activity

## Abstract

Considering interactions between barriers to physical activity, sociodemographic factors, and rurality can support an equity-focused approach to physical activity promotion. In this cross-sectional analysis of the Canadian Community Health Survey Barriers to Physical Activity Rapid Response module, we compared self-reported individual and social-environmental correlates of physical activity between rural and urban residents and explored interactions with sociodemographic factors. Lack of social support was associated with lower odds of meeting physical activity guidelines for rural residents (OR = 0.71 [0.57,0.89], p = 0.003), but not for urban residents (OR = 0.99 [0.84,1.17], p =.931). Limited access to low-cost facilities was associated with lower odds of meeting physical activity guidelines (OR = 0.85 [0.73,0.98], p = 0.030) regardless of location, but was reported more commonly as a barrier by rural males (27.3 % vs 8.6 % urban) and females (30.0 % vs 9.1 % urban). Inadequate social support was associated with lower odds of meeting physical activity guidelines in females (OR = 0.79 [0.66,0.94], p =.009), but not males (OR = 0.99 [0.84,1.17], p =.931). Individual-level barriers such as time, costs, enjoyment, and confidence were associated with meeting physical activity guidelines for both rural and urban residents. Social-environmental factors appear to be the main contributors to physical activity inequities between rural and urban residents. Interventions designed to bolster social connectedness may support physical activity engagement for people living in rural communities.

## Highlights

• Barriers related to social support were associated with less physical activity in rural residents.
• Lack of social support is a barrier to physical activity for females in rural and urban locations.
• Access to facilities is associated with meeting physical activity guidelines for both rural and urban residents.
• Individual level barriers impact physical activity similarly for rural and urban residents.

## 1 Introduction

Regular participation in physical activity is important for overall well-being including noncommunicable disease prevention, social connectedness, and mental health (Chekroud et al., 2018, Lee et al., 2012, Penedo and Dahn, 2005). Despite well-established benefits, there are substantial inter-individual variations in physical activity behavior which are associated with sociodemographic (e.g., gender, age, and socioeconomic status; Trost et al., 2002, Plotnikoff et al., 2004, Abichahine and Veenstra, 2017) and environmental factors (e.g., rurality; Pelletier et al., 2021a, Lim et al., 2021). Unequal distribution of opportunities for engagement in physical activity and for physical activity promotion contribute to inequalities in physical activity behavior (Chastin et al., 2020, Althoff et al., 2017, Cleland et al., 2010). Designing and implementing equitable physical activity interventions necessitates a comprehensive understanding of how multifaceted factors interact to generate unequal opportunities between individuals and groups (Bauer, 2014).

Through the lens of a social-ecological model, physical activity correlates (i.e., factors associated with physical activity in cross-sectional analyses), can be classified at individual, interpersonal, environmental, and policy levels (Bauman et al., 2012). Individual correlates of physical activity include preferences for activity, self-efficacy, cost, education, income, sex, gender, and age (Saelens et al., 2012, Pelletier et al., 2021a, Bauman et al., 2012). Built environmental correlates include the availability of green space, access to recreation facilities, and road connectivity (McCormack and Shiell, 2011, Saelens and Handy, 2008, Astell-Burt et al., 2014). The social-environmental factors associated with physical activity include sense of community cohesion and belonging, perceptions of safety, and social support (Samuel et al., 2014).

For rural adults living in high-income countries, barriers to physical activity include distance to facilities, built environmental factors (e.g., lack of sidewalks), and a lower tax-base leading to inadequate funding for recreation (Badland et al., 2008, Huang et al., 2018, Frost et al., 2010). Rural communities also have a distinct sociocultural environment of physical activity (e.g., conceptualization of physical activity types and purpose of activity; Barnidge et al., 2013) and a different sociodemographic profile (e.g., older age, lower educational attainment) compared to urban residents (Pelletier et al., 2021a).

Place, or geographical location, must be considered as a variable in intersectional approaches to understanding health behavior (Bambra, 2022). While evidence suggests lower physical activity participation among rural adults (Fan et al., 2014, Martin et al., 2005), sex and gender have been identified as moderators in the relationship between geographical location and physical activity (Bengoechea et al., 2005, Pelletier et al., 2021a). As previous work exploring how multiple intersecting factors impact physical activity behavior in Canada has not included rurality as a factor (Abichahine and Veenstra, 2017), exploring interactions between rural–urban location and sociodemographic factors will advance understanding of physical activity correlates in high-income countries.

Most physical activity epidemiological and behavioral science studies conducted in Canada or similar high-income countries focus on urban environments (e.g., Pan et al., 2009) or exclusively on rural sub-populations without a robust urban comparison (e.g., Solomon et al., 2013, Chrisman et al., 2014, Cleland et al., 2012) which limits understanding of how to tailor context-specific active living strategies to rural communities (Umstattd Meyer et al., 2016, Barnidge et al., 2013). As definitions of rurality vary considerably across jurisdictions both within and across countries (Gessert et al., 2015, Hart et al., 2005, Bennett et al., 2019), country-specific nationally representative analyses are required to capture interactions between socioeconomic, demographic, and place-based variables.

This study aims to: a) compare the relationship between individual and environmental barriers as correlates of physical activity in rural and urban areas of Canada; and b) explore interactions between sociodemographic factors and individual and environmental correlates of behavior based on rural–urban location.

## 2 Methods

### 2.1 Data sources

Data used for this cross-sectional analysis are from the Canadian Community Health Survey (CCHS) 2017 annual cycle and the Barriers to Physical Activity Rapid Response as described in our previous work (Pelletier et al., 2021a, Pelletier et al., 2021b). The CCHS is an annual survey recording self-reported health status and health behaviors of adults and youth living in Canada. The survey provides a nationally representative sample based on complex sampling and weighting strategies (Statistics Canada, 2018). The survey is completed by one member of each selected household using computer-assisted interviewing (telephone or in person) by trained interviewers across four data collection periods (January to March, April to June, July to September, October to December) as described in the CCHS user guide (Statistics Canada, 2018). The Barriers to Physical Activity Rapid Response was added to the 2017 CCHS annual cycle during the July to September and October to December collection periods. The 2017 Barriers to Physical Activity Rapid Response module had a combined Canada-level response rate of 61.7 % (Statistics Canada, 2018). We excluded youth participants (age < 18, n = 2149) prior to any examination of the data. We also excluded adults who reported currently being pregnant or who did not answer (n = 296), and individuals with missing data (refusal or not stated responses) on any variable of interest (n = 3611; see supplementary file Table 1a). Our final population-weighted sample was n = 24,499,462 (unweighted n = 21,967). Each respondent provided consent before completing the CCHS. All data were accessed and vetted for release following Statistics Canada regulations designed to protect respondent privacy and ensure no data are identifiable.

### 2.2 Variables

The CCHS variables and definitions used in this analysis are available in Table 1.

**Table 1:** Canadian Community Health Survey Barriers to Physical Activity Rapid Response variables used in analysis and regression coding.

CCHS Variable	Definition	Regression coding
PAADVMVA	A measure of physical activity relative to the Canadian Physical Activity Guidelines	Collapsed to binary <150/≥150 min per week 0: <150 min/week
GEODVUR2	A dichotomous indicator of rurality categorized location as urban or rural.	0: Urban
HWTDVCOR	Body mass index derived from self-report height and weight.	Continuous mean-centered
EHG2DVR3	Education was measured using a three-level variable: less than secondary school graduation/secondary school graduation/post-secondary certificate, diploma, or university degree)	0: secondary school graduation
INCDVHH	Household income self-reported with 15 levels	Collapsed to 5-level weighted quintiles 0: $60–90,000/year
GENDVHDI	Self-reported perceived health status, self-reported - very poor to excellent.	0: Good
GEN_030	Sense of belonging to community, self-reported with four levels from very strong to very weak.	0: Very weak
DHH_AGE	Age	Continuous mean-centered
DHH_SEX	Self-reported sex (male or female)	0: Male
SAM_CP	Sample collection period (season of data collection) – sample was divided into July to September and October to December.	0: July–September

CCHS, Canadian Community Health Survey.

**Physical activity.** The physical activity variable was derived from 11 items asking about weekly minutes of active transportation, moderate-to-vigorous recreational activity, and moderate-to-vigorous occupational and household-related physical activity. Reported minutes of activity in the past 7 days across three domains was combined to produce the 3-level physical activity variable used for this analysis. Physical activity was measured with a derived variable calculated based on self-reported minutes per day of physical activity aggregated into categories: 0 min of moderate-to-vigorous physical activity (MVPA) per week, 1–149 min of MPVA per week, and ≥150 min of MVPA per week across all domains of activity (transportation, sport/fitness or leisure, occupational/household). We collapsed these three categories into a binary variable indicating whether participants engaged in <150 min per week or ≥150 min per week of MVPA to reflect meeting Physical Activity Guidelines (Bull et al., 2020). This variable was employed as the dependent variable in all regression analyses.

**Rural-urban location.** The CCHS provides three variables specifying rural/urban location at different levels of resolution, from binary rural/urban designation up to a 7-level variable designating rural, metropolitan fringe, and metropolitan core areas. All geographic variables are derived from the respondents’ postal code. Preliminary comparisons showed no differences in outcomes of interest as a function of different location designations. For simplicity, geographic data was taken from a binary measure of rural/urban location. A population centre (urban) was defined by Statistics Canada as having a minimum population concentration of 1000 with a population density of at least 400 people per square kilometre, while a rural area was defined by a population of fewer than 1000 people or a population density below 400 people per square kilometre.

**Sociodemographic variables.** Demographic information included sex, age, body mass index (BMI), education, and household income. We included a measure of perceived health status, sense of belonging to the community, and season of data collection as described in our previous work (Pelletier et al., 2021b).

**Individual and social-environmental barriers to physical activity.** Participants were asked to rate their agreement with statements related to individual (6 items) and social-environmental (3 items) barriers to physical activity from 1 = strongly agree to 4 = strongly disagree (Table 2). Items were recoded into numeric binary variables, collapsing “Strongly Agree” and “Agree” (coded as 0 for no barrier reported), and “Disagree” and “Strongly Disagree” (coded as 1 for barrier reported). Based on the social-ecological model (Sallis et al., 2015, McLeroy et al., 1988) and our previous work (Pelletier et al., 2021b), we combined these items into three general barrier domains coded as binary variables (0 = no barrier reported; 1 = any barrier reported).

**Table 2:** Prevalence of reporting barriers items measured in the Barriers to Physical Activity Rapid Response.

Barrier domains	Barrier items		Male (n = 12343443)				Female (n = 12156019)			
			Urban (n = 10,270,509)		Rural (n = 2,072,934)		Urban (n = 10,019,945)		Rural (n = 2,136,074)	
			N	%	N	%	N	%	N	%
Individual motivation-related barriers	I prefer to be physically active rather than sitting or lying down	no barrier	9,262,609	90.2	1,931,189	93.2	8,696,982	86.8	1,922,305	90.0
		barrier	1,007,900	9.8	141,745	6.8	1,322,963	13.2	213,769	10.0
	I am confident in my ability to engage in physical activity	no barrier	9,692,523	94.4	1,942,902	93.7	9,058,670	90.4	1,963,575	91.9
		barrier	577,986	5.6	130,032	6.3	961,275	9.6	172,499	8.1
	I enjoy being physically active	no barrier	9,689,879	94.3	1,989,175	96.0	9,187,877	91.7	1,978,437	92.6
		barrier	580,630	5.7	83,759	4.0	832,068	8.3	157,637	7.4

Individual resource-related variables	I have enough energy to be physically active on a regular basis	no barrier	9,398,226	91.5	1,905,211	91.9	8,387,309	83.7	1,830,151	85.7
		barrier	872,283	8.5	167,723	8.1	1,632,637	16.3	305,923	14.3
	I have enough time to be physically active on a regular basis	no barrier	8,152,784	79.4	1,701,822	82.1	7,717,997	77.0	1,683,907	78.8
		barrier	2,117,724	20.6	371,112	17.9	2,301,949	23.0	452,168	21.2
	I can afford the costs of being physically active on a regular basis	no barrier	8,911,271	86.8	1,794,506	86.6	8,000,286	79.8	1,727,698	80.9
		barrier	1,359,238	13.2	278,428	13.4	2,019,659	20.2	408,376	19.1

Social-environmental barriers	I often see people in my community being physically active	no barrier	8,722,844	84.9	1,714,591	82.7	8,774,700	87.6	1,805,617	84.5
		barrier	1,547,665	15.1	358,343	17.3	1,245,245	12.4	330,457	15.5
	My neighbourhood has several free or low-cost recreation facilities, such as parks, walking trails, bike paths, recreation centres, playgrounds or public swimming pools	no barrier	9,385,343	91.4	1,507,910	72.7	9,107,714	90.9	1,494,902	70.0
		barrier	885,165	8.6	565,023	27.3	912,231	9.1	641,172	30.0
	I receive support to be physically active on a regular basis from friends, family members or other people in my life	no barrier	7,908,324	77.0	1,496,407	72.2	7,925,532	79.1	1,618,009	75.7
		barrier	2,362,185	23.0	576,526	27.8	2,094,414	20.9	518,066	24.3

### 2.3 Data analysis

Data analysis was conducted with R 3.4.3 (R Core Team, 2019) using packages arsenal (Heinzen et al., 2019) and survey (Lumley, 2019). All analyses employed survey weights and bootstrap replicate resampling weights provided by Statistics Canada to ensure outcomes were representative of the Canadian population and to account for the complex sampling design of the CCHS.

We examined how self-reported barriers to physical activity were associated with the likelihood of meeting Canadian physical activity guidelines (e.g., as correlates of behavior) using binomial logistic regression and adjusting for sociodemographic factors. A stepped model-fitting approach was used to determine the best-fitting model. For each outcome, we first computed a base model to assess the association between urban/rural location and the likelihood of meeting physical activity guidelines. Next, a model was computed entering the three barrier domains (individual resource-related, individual motivation-related, and social-environmental) together as predictors.^2^ All sociodemographic covariates were added to the model, after which we sequentially removed sociodemographic covariates not contributing to explaining significant variance in the overall model. Variables were removed in order of smallest *t*-value until none could be further removed without reducing explanatory power according to log-likelihood ratio comparisons of model fit. Given study aims, the location factor was retained regardless of contribution to the model.

After computing the best-fitting covariate model, we estimated 2-way interactions between location and barrier domains, and an *a priori-*determined sex X location interaction based on our previous findings (Pelletier et al., 2021a). If there was more than one 2-way interaction with location, we estimated 3-way interaction terms. Wherever a barrier domain contributed to the model, the final step in our model fitting involved replacing the domain factor with the individual items to improve resolution in characterizing associations between self-reported barriers and meeting physical activity guidelines. Barrier item X location interactions were only tested if the overall barrier domain interacted with location to reduce the overall number of tests. All barrier items were also tested in interaction with sex given the importance of sex as a moderating variable in previous work. For brevity only significant interactions are reported.

## 3 Results

### 3.1 Participants

Participant demographics based on rural/urban location are summarized in Table 3.

**Table 3:** Population-weighted demographics by rural–urban location.

Variables of interest		Rural (N = 4,209,008)		Urban (N = 20,290,454)		Total (N = 24,499,462)		p-value
		N	%/SE	N	%/SE	N	%/SE	
Season	Summer	2,012,891	47.8	10,218,419	50.4	12,231,310	49.9	0.262
	Fall	2,196,117	52.2	10,072,035	49.6	12,268,152	50.1	

Sex	Male	2,072,934	49.2	10,270,509	50.6	12,343,443	50.4	0.282
	Female	2,136,074	50.8	10,019,945	49.4	12,156,019	49.6	

Age	Mean	50.6	0.354	46.1	0.138	46.9	0.102	< 0.001
	95 % CI	[49.9, 51.3]		[45.8, 46.3]		[46.7, 47.1]		

BMI	Mean	28.3	0.137	27.2	0.074	27.4	0.068	< 0.001
	95 % CI	[28.0, 28.5]		[27.1, 27.4]		[27.3, 27.5]		

Meet PA guidelines	No	1,755,912	41.7	7,740,793	38.1	9,496,705	38.8	0.006
	Yes	2,453,095	58.3	12,549,661	61.9	15,002,756	61.2	

Education	Less than high school	677597.1	16.1	1731009.5	8.5	2408606.6	9.8	< 0.001
	High school	1206112.2	28.7	5,033,644	24.8	6239756.2	25.5	
	Post-secondary	2325298.4	55.2	13525800.5	66.7	15851098.9	64.7	

Income	$0–29,999	485665.7	11.5	2563632.1	12.6	3049297.8	12.4	< 0.001
	$30–59,999	942434.9	22.4	4,041,778	19.9	4984212.9	20.3	
	$60–99,999	1126413.9	26.8	4927414.2	24.3	6053828.1	24.7	
	$100–149,999	899355.1	21.4	4251121.1	21.0	5150476.2	21.0	
	$150,000+	755,138	17.9	4506508.5	22.2	5261646.5	21.5	

Perceived Health	Excellent	1032096.9	24.5	4,968,586	24.5	6000682.9	24.5	0.02
	Very good	1565836.6	37.2	7808784.6	38.5	9374621.2	38.3	
	Good	1145131.2	27.2	5649887.1	27.8	6795018.3	27.7	
	Fair	347232.5	8.2	1475061.9	7.3	1822294.4	7.4	
	Poor	118710.4	2.8	388134.4	1.9	506844.8	2.1	

Sense of Belonging to Community	Very Strong	854929.3	20.3	3281955.4	16.2	4136884.7	16.9	< 0.001
	Somewhat strong	2,077,782	49.4	10527191.8	51.9	12604973.8	51.5	
	Somewhat weak	988478.6	23.5	5063461.5	25.0	6051940.1	24.7	
	Very Weak	287817.7	6.8	1417845.3	7.0	1,705,663	7.0	

P-value obtained from *t*-test or Chi-square test as appropriate.

As reported in: Pelletier, C. A., White, N., Duchesne, A., Sluggett, L. 2021. Barriers to physical activity for adults in rural and urban Canada: A cross-sectional comparison. *SSM-Popul. Health*, 100964. https://doi.org/10.1016/j.ssmph.2021.100964.

### 3.2 Regression models

**Fig. 1:**
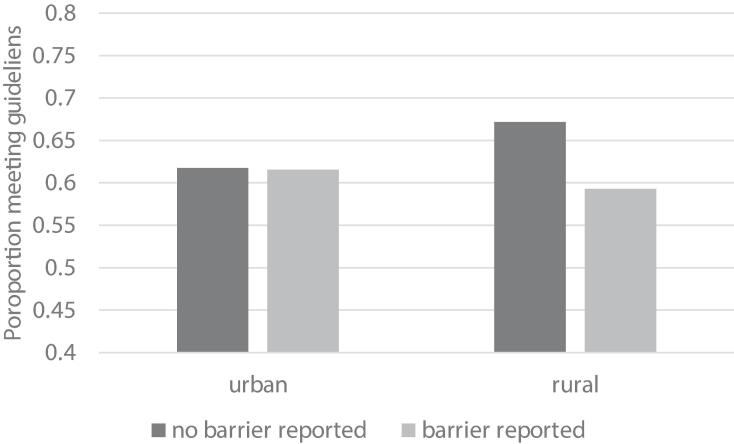
Interaction between rural–urban location and social support on meeting physical activity guidelines. Note: for clarity logit values have been back transformed to percent of sample reporting.

In the base model examining the effect of location alone, rural residents were less likely than urban residents to meet physical activity guidelines (OR = 0.86, 95 % CI [0.78, 0.96], *p* =.006) (Fig. 1).

***Models with barrier domains:*** Across all domains, reporting at least one barrier was associated with significantly lower odds of meeting physical activity guidelines (Table 4). In the optimally-fitted model (supplementary file Table 2A), the main effect of location was significant, with rural residents showing *higher* odds of meeting activity guidelines compared to urban residents (OR = 1.33 [1.10, 1.60]); this effect of location was qualified by two significant interactions. One was a significant sex X location interaction (OR = 0.70 [0.56, 0.86], *p* =.001). The second was an interaction between the domain of social-environmental barriers and location (OR = 0.73 [0.59, 0.91], *p* =.004; see Table 4). This interaction is further expanded below in the item-level analysis.

**Table 4:** Odds of meeting physical activity guidelines when reporting barriers to physical activity (domain level) in step 2 (barrier domains + location) and optimally-fitted models.

	Step 2 model	Optimally-fitted model
Individual motivation-related	OR = 0.41 [0.36, 0.47], *p* < .0001	OR = 0.58 [0.50, 0.67], *p* < .0001
Individual resource-related	OR = 0.63 [0.57, 0.71], *p* < .0001	OR = 0.51 [0.45, 0.57], *p* < .0001
Social-environmental	OR = 0.83 [0.75, 0.91], *p* = .0001	**Urban**: OR = 0.96 [0.86, 1.08], *p* = .088 **Rural**: OR = 0.71 [0.59, 0.85], p = .0002

***Models with barrier items****:* In the final model with barrier items (supplementary file Table 3A), the sex X location interaction remained significant (OR = 0.70 [0.56, 0.86], *p* =.0009). For males, living in a rural area was associated with significantly higher odds of meeting recommended physical activity guidelines (OR = 1.27 [1.07, 1.50], *p* =.006), while no effect of location was observed for females (OR = 0.88 [0.74, 1.04], *p* =.143). For urban residents there was no effect of sex in relation to physical activity (OR = 1.01 [0.88, 1.15], *p* =.910) while for rural residents, females demonstrated significantly lower odds of meeting activity guidelines compared to males (OR = 0.70 [0.58, 0.85], *p* =.0002).

Individual resource- and motivation-related items were significantly associated with meeting physical activity guidelines but were not tested in interaction with rural–urban location due to non-significant interactions at the domain level (Table 5). Regarding social-environmental items, we observed a significant support X location interaction (OR = 0.72 [0.57, 0.91], *p* =.006). Rural residents with adequate social support to be active showed significantly *higher* odds of meeting physical activity guidelines (OR = 1.27 [1.07, 1.50], *p* =.006) compared to urban residents. However, rural residents who lacked social support were significantly less likely to meet activity guidelines (OR = 0.71 [0.57, 0.89], *p* =.003) compared to those with adequate social support. In contrast, there was no association between social support and the likelihood of meeting activity guidelines for urban residents (OR = 0.99 [0.84, 1.17], *p* =.931).

**Table 5:** Odds of meeting physical activity guidelines when reporting barrier items in optimally fitted models.

**Barrier Item**	**Optimally-fitted model**	**Interactions**
**Individual motivation-related items**		
I prefer to be physically active rather than sitting or lying down	OR = 0.54 [0.45, 0.64], *p* < .0001	Not tested in interaction with location due to non-significant interaction at domain level.
I am confident in my ability to engage in physical activity	OR = 0.62 [0.50, 0.76], *p* < .0001	
I enjoy being physically active	OR = 0.72 [0.58, 0.89], *p* = .002	

**Individual resource-related items**		
I have enough energy to be physically active on a regular basis	OR = 0.79 [0.67, 0.94], *p* = .008	Not tested in interaction with location due to non-significant interaction at domain level.
I have enough time to be physically active on a regular basis	OR = 0.55 [0.48, 0.63], *p* <. 0001	
I can afford the costs of being physically active on a regular basis	OR = 0.95 [0.82, 1.10], *p* = .493	

**Social-environmental items**		
I often see people in my community being physically active	OR = 1.21 [1.04, 1.41], *p* = .016	No significant interaction with rural–urban location
I receive support to be physically active on a regular basis from friends, family members or other people in my life	Not reported due to significant interaction with location	**Support × location** Urban: OR = 0.99 [0.84, 1.17], *p* = .931 Rural: OR = 0.71 [0.57, 0.89], *p* = .003 **Support × sex** Males: OR = 0.99 [0.84, 1.17], *p* = .931 Females: OR = 0.79 [0.66, 0.94], *p* = .009
My neighbourhood has several free or low-cost recreation facilities, such as parks, walking trails, bike paths, recreation centres, playgrounds or public swimming pools	OR = 0.85 [0.73, 0.98], *p* = .030	No significant interaction with rural–urban location

**Fig. 2:**
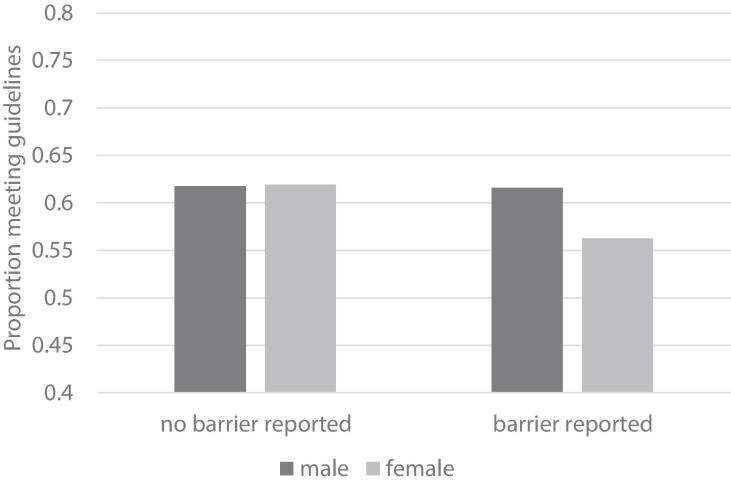
Interaction between self-identified sex and social support on meeting physical activity guidelines. Note: for clarity logit values have been back transformed to percent of sample reporting.

We observed a significant support X sex interaction (OR = 0.80 [0.64, 0.99], *p* =.042, Fig. 2). For males, there was no association between social support and meeting physical activity guidelines (OR = 0.99 [0.84, 1.17], *p* =.931). For females, lack of social support was associated with significantly lower odds of meeting guidelines (OR = 0.79 [0.66, 0.94], *p* =.009). When no social support barriers were reported, males and females did not differ in the odds of meeting activity guidelines (OR = 1.01 [0.88, 1.15], *p* =.910). When lack of social support was reported, females were significantly less likely to meet activity guidelines compared to males (OR = 0.80 [0.65, 0.99], *p* =.037). The addition of a three-way sex X support X location interaction term did not significantly improve model fit (X^2^(1) = 5.07, *p* =.08).

## 4 Discussion

We examined the association between barriers to physical activity and the odds of meeting physical activity guidelines by rural–urban geographical location. Additionally, we examined whether sex and self-reported barriers moderated the relationship between rural–urban location and physical activity. While individual-level barriers were significantly associated with meeting physical activity guidelines, they did not interact with rural–urban location. This finding suggests geographical location is not associated with how people perceive the impact of having enough time, energy, and financial resources to be physically active on a regular basis.

Lack of social support was not associated with meeting physical activity guidelines for urban residents. In rural residents, lack of social support was more commonly reported and was associated with significantly lower odds of meeting physical activity guidelines. The role of social support and social norms may be important factors for understanding physical activity behavior in rural communities. Rural residents often identify social engagement as the main facilitator of physical activity participation and describe physical activity as a way to connect with their communities (Cleland et al., 2015). Similar work identified social support as an important factor related to physical activity for rural adults in the United States (Cleland et al., 2010, Eyler, 2003), however, in some studies, social support was only important for the sport domain of physical activity (Chrisman et al., 2014). Our observed sex X support interaction identifies reporting a lack of social support has a stronger association with physical activity behavior for females than males. Several papers have similarly identified the importance of social support for women to engage in physical activity, where support is often related to caring responsibilities, social resources, and comfort accessing exercise facilities (Cleland et al., 2012; Van Dyck et al., 2014, Scarapicchia et al., 2017, Morris et al., 2020, Vrazel et al., 2008, Wilson et al., 2022). Reduced gendered responsibilities (e.g., childcare, household tasks) are associated with greater odds of meeting physical activity guidelines for rural women (Eyler, 2003), and tailored interventions to address gender-specific barriers are effective at increasing physical activity for women (Segar et al., 2002). There appears to be a unique sociocultural role of gender in rural settings and a need for social support to facilitate participation in physical activity for rural women. Our findings are a novel advancement to prior work in identifying interactions between sex, social support, and rurality although we note our analysis was limited by a self-reported binary measure of sex rather than gender. Further, while we recognize an intersectional approach to gender might provide a more accurate depiction of how gender is being enacted in relation to physical activity, our current analysis did not demonstrate interactions across multiple social categories (e.g., sex X location X support). Exploring how gender influences and interacts with socioeconomic factors and social-ecological barriers to physical activity for rural and urban dwelling women and gender diverse people in Canada is an important area for future study.

We previously reported rural residents were four times more likely than urban residents to identify lack of facility access as a barrier to physical activity (Pelletier et al, 2021b). In the current analysis, participants reporting barriers related to facility access were less likely to meet physical activity guidelines. However, we did not observe an interaction between lack of facility access and rural–urban location in predicting the likelihood of meeting physical activity guidelines. This finding suggests a lack of access to facilities is associated with less physical activity engagement regardless of location (i.e., people who live in either a rural or urban location without access to spaces to be active are less likely to meet physical activity guidelines). All the same, given rural residents are far more likely to report limited access to facilities, and access is associated with meeting guidelines, increasing access to low-cost nearby facilities in rural areas may be an important strategy to support physical activity.

Proximity to recreation facilities has an inconsistent association with walking and light physical activity, although the presence of facilities has a positive association with physical activity more broadly (Sawyer et al., 2017a, Eriksson et al., 2012, Solomon et al., 2013). Among rural women, the only physical environmental correlate of activity identified is adequate street lighting, with facility access found to be a non-significant predictor (Eyler, 2003). Given proximity to outdoor recreation is a facilitator of physical activity for rural residents, it is possible people in rural communities perceive less access to low-cost facilities but manage to maintain their activity independently of traditional exercise facilities. As individual-level variables including intention, confidence, and enjoyment are identified as the dominant determinants of physical activity behavior, it is also possible a lack of facility access was identified by survey participants but ultimately did not impact behavior – particularly in cases where individuals have high individual motivation or intentions to be active (Rhodes et al., 2017, Bauman et al., 2012). Considering physical activity as a behavior within a complex system, more work is needed to understand how individual variables (social, cognitive and demographic) interact with opportunities to impact behavior (Sniehotta et al., 2017) while considering interacting social categories (i.e., intersectionality; Lim et al., 2021).

Future work should additionally explore neighborhood-level factors of the built and natural environment across the rural–urban continuum, including survey questions or direct geographical measurements (e.g., GIS, proximity to walking trails or greenspace) to comprehensively capture active living environments (Butler et al., 2011, Brownson et al., 2009, Kajosaari and Laatikainen, 2020). Our physical activity outcome variable represents a combination of MVPA across all domains, and it is possible the relationship between the built environment and physical activity may differ based on the specific domain of interest (Sallis et al., 2015, Sawyer et al., 2017b). An area of focus for future work is to explore how domain of physical activity measured varies by socioeconomic status, sex and gender, and rurality.

Our study provides a comparison of the correlates of physical activity between adults living in rural and urban communities in Canada. While these findings are based on a nationally representative sample, the context of rurality and how it influences physical activity engagement are likely mostly applicable to Canada and other high-income countries. Our findings may not be generalizable to low- or middle-income countries. Future work should consider country-level economic status as part of an intersectional approach to understanding physical activity behavior.

## 5 Limitations

This study is cross-sectional and does not provide a prospective analysis of causal relationships (e.g., determinants of behavior). Like our previous work (Pelletier et al., 2021b), we note differences between included and excluded samples. As such, our analysis is representative of the Canadian population for whom complete data were available (i.e., not representative of the full Canadian population). Excluded participants were more likely to be female and less likely to meet physical activity guidelines than the included sample, among other differences (see Supplementary files). This reflects possible biases in terms of survey completion. Our included sample generally reflects participants with higher socioeconomic status and physical activity participation than the wider Canadian population and for whom the impact of barriers to physical activity (e.g., costs, access) may be less limiting on physical activity. Self-report of barriers to physical activity may not provide as accurate a picture of physical activity inequalities as direct measures (e.g., neighborhood characteristics), but are still useful in highlighting people’s perceptions of these issues (e.g., perceived access to facilities). The study was also limited by the items included in the Barriers to Physical Activity Rapid Response, particularly related to environmental barriers. The survey included no questions regarding the role of the natural environment, which has been shown to be an important factor impacting physical activity participation for rural communities (Abildso et al., 2021).

## 6 Conclusions

In a nationally representative Canadian sample, we have identified social-environmental factors as one of the main influences on physical activity inequities between rural and urban residents. As lack of social support appears to be particularly important for females, exploring strategies to bolster social support though a sex/gender lens may represent an equity-driven physical activity promotion strategy. It is essential to consider an intersectional approach to understanding and supporting physical activity behavior by considering how sex and gender interact with other social categories (e.g., income, emotional support, rurality). Individual barriers were related to physical activity behavior but did not differ based on rural–urban location and are appropriate targets for an all-population approach to physical activity promotion.

## Funding

This study was funded by a Research Data Centre award from the University of Northern British Columbia.

## CRediT authorship contribution statement

**Chelsea Pelletier:** Conceptualization, Funding acquisition, Methodology, Writing – original draft. **Nicole White:** Data curation, Formal analysis, Methodology, Visualization, Writing – original draft. **Annie Duchesne:** Conceptualization, Funding acquisition, Methodology, Writing – review & editing. **Larine Sluggett:** Conceptualization, Methodology, Writing – review & editing.

## Declaration of Competing Interest

The authors declare that they have no known competing financial interests or personal relationships that could have appeared to influence the work reported in this paper.

## Data Availability

The authors do not have permission to share data.

## Footnotes

2 We use the term “predictor” with respect to its statistical usage within the scope of regression model outcomes for our cross-sectional analysis. Directionality of relationships between variables and/or their causal relations to one another cannot be assessed. We do not advance any causal interpretations in the present analysis.
