# Supplementary material for: Rural-urban differences in individual and environmental correlates of physical activity in Canadian adults
Source: Prev Med Rep. 2022 Nov 21;30:102061. doi: 10.1016/j.pmedr.2022.102061 (PMC9747644; doi:10.1016/j.pmedr.2022.102061)
Supplement: Supplementary data 1 [file mmc1.docx]

Table 1A. Population-weighted demographics for included vs. excluded participants

| **Variables of interest** | | **Excluded (N=4331267.6)** | | **Included (N=24499461.6)** | | **Total (N=28,830,729.2)** | | **p-value** |
| --- | --- | --- | --- | --- | --- | --- | --- | --- |
|  |  | **N** | **% / SE** | **N** | **% / SE** | **N** | **% / SE** |  |
| **Location** | **Urban** | 3628699.7 | 83.8 | 20290454 | 82.8 | 23919153.7 | 83.0 | 0.271 |
|  | **Rural** | 702567.9 | 16.2 | 4209007.6 | 17.2 | 4911575.5 | 17.0 |  |
| **Season** | **Summer** | 2171827 | 50.1 | 12231310 | 49.9 | 14403137 | 50.0 | 0.889 |
|  | **Fall** | 2159441 | 49.9 | 12268152 | 50.1 | 14427593 | 50.0 |  |
| **Sex** | **Male** | 1837785 | 42.4 | 12343442 | 50.4 | 14181227 | 49.2 | < .001 |
|  | **Female** | 2493482 | 57.6 | 12156019 | 49.6 | 14649501 | 50.8 |  |
| **Age** | **Mean (SE)** | 54.4 | 0.5 | 46.8 | 0.1 | 48 | 0.1 | < .001 |
|  | **95% CI** | [53.4, 55.5] |  | [46.7, 47.1] |  | [47. 9, 48.1] |  |  |
| **BMI** | **Mean (SE)** | 27.6 | 0.2 | 27.4 | 0.1 | 27.4 | 0.1 | 0.39 |
|  | **95% CI** | [27.2, 27.9] |  | [27.3, 27.5] |  | [27.3, 27.5] |  |  |
| **Education** | **Less than high school** | 788053.8 | 20.4 | 2455527.2 | 9.8 | 3243581 | 11.3 | < .001 |
|  | **High school** | 1003997.4 | 26.1 | 6361309.1 | 25.5 | 7365306.5 | 25.5 |  |
|  | **Post-secondary** | 2061957.2 | 53.5 | 16159884.5 | 64.7 | 18221841.7 | 63.2 |  |
| **Income** | **$0-29,999** | 765471.3 | 17.7 | 3049297.8 | 12.4 | 3814769.1 | 13.2 | < .001 |
|  | **$30-59,999** | 1189721.1 | 27.5 | 4984212.9 | 20.3 | 6173934 | 21.4 |  |
|  | **$60-99,999** | 1020559.8 | 23.6 | 6053828.1 | 24.7 | 7074387.9 | 24.5 |  |
|  | **$100-149,999** | 722784.9 | 16.7 | 5150476.2 | 21.0 | 5873261.1 | 20.4 |  |
|  | **$150,000+** | 632730.5 | 14.6 | 5261646.6 | 21.5 | 5894377.1 | 20.4 |  |
| **Perceived Health** | **Excellent** | 339047.4 | 7.9 | 507274.4 | 2.1 | 846321.8 | 2.9 | < .001 |
|  | **Very good** | 593031.3 | 13.8 | 1823839 | 7.4 | 2416870.3 | 8.4 |  |
|  | **Good** | 1392037.4 | 32.3 | 6800777.6 | 27.7 | 8192815 | 28.4 |  |
|  | **Fair** | 1170851.4 | 27.2 | 9382566.9 | 38.3 | 10553418.3 | 36.6 |  |
|  | **Poor** | 815534.9 | 18.9 | 6005768.8 | 24.5 | 6821303.7 | 23.7 |  |
| **Sense of Belonging to Community** | **Very Strong** | 395390.7 | 12.2 | 1781127.6 | 7.0 | 2176518.3 | 7.5 | < .001 |
|  | **Somewhat strong** | 692068.2 | 21.3 | 6319699.4 | 24.7 | 7011767.6 | 24.3 |  |
|  | **Somewhat weak** | 1578679.5 | 48.6 | 13162662.4 | 51.5 | 14741341.9 | 51.1 |  |
|  | **Very Weak** | 581186.4 | 17.9 | 4319915.1 | 16.9 | 4901101.5 | 17.0 |  |

- P-value obtained from t-test or Chi-square test as appropriate
- Note: total included ns for individual self-report variables may sum to greater than the sample-wide included N due to participants missing data on some but not all items. All participants with missing data on any item were marked for sample-wide exclusion.

As reported in: Pelletier, C. A., White, N., Duchesne, A., & Sluggett, L. (2021). Barriers to physical activity for adults in rural and urban Canada: A cross-sectional comparison. *SSM-Population Health*, 100964. https://doi.org/10.1016/j.ssmph.2021.100964

Table 2A. Optimally fitted barrier domains model including sociodemographic covariates and significant interaction terms

| Coefficients: | Odds | lower ci | upper ci | t value | Pr(>\|t\|) | Sig. |
| --- | --- | --- | --- | --- | --- | --- |
| (Intercept) | 1.733 | 1.453 | 2.067 | 6.108 | <.0001 | *** |
| Age | 0.980 | 0.977 | 0.982 | -14.031 | <.0001 | *** |
| BMI | 1.013 | 1.004 | 1.023 | 2.695 | 0.0072 | ** |
| Less than high school | 0.799 | 0.669 | 0.957 | -244.10 | 0.0148 | * |
| Post-secondary edu | 1.241 | 1.095 | 1.406 | 3.373 | 0.0008 | *** |
| Income$0-29k | 0.774 | 0.656 | 0.914 | -3.028 | 0.0025 | ** |
| Income$30-59.9k | 0.782 | 0.671 | 0.912 | -3.134 | 0.0018 | ** |
| Income$100-150k | 0.941 | 0.811 | 1.091 | -0.808 | 0.4193 |  |
| Income$150k+ | 1.128 | 0.967 | 1.316 | 1.538 | 0.1244 |  |
| PerceivedHealthVeryPoor | 0.841 | 0.620 | 1.142 | -1.107 | 0.2686 |  |
| PerceivedHealthPoor | 1.120 | 0.930 | 1.349 | 1.193 | 0.2331 |  |
| PerceivedHealthVeryGood | 1.274 | 1.115 | 1.456 | 3.551 | 0.0004 | *** |
| PerceivedHealthExcellent | 1.333 | 1.144 | 1.553 | 3.678 | 0.0002 | *** |
| IndivMotivationBarrier | 0.577 | 0.495 | 0.672 | -7.063 | <.0001 | *** |
| IndivResourceBarrier | 0.506 | 0.445 | 0.574 | -10.478 | <.0001 | *** |
| SexFemale | 0.951 | 0.842 | 1.073 | -0.823 | 0.4107 |  |
| LocationRural | 1.328 | 1.105 | 1.596 | 3.023 | 0.0026 | ** |
| EnviroBarrier | 0.963 | 0.859 | 1.080 | -0.638 | 0.5239 |  |
| Sex:Location | 0.695 | 0.560 | 0.862 | -3.316 | 0.0009 | *** |
| Location:EnviroBarrier | 0.733 | 0.590 | 0.910 | -2.819 | 0.0049 | ** |

NOTE: Intercept reflects odds reflecting the intersection of all regression anchors and as such the level of each variable serving as the regression anchor does not appear in the list of coefficients. See Table 1 for coding strategy.

*p<*.05 = * ; *p*<.01 = ** ; *p*<.0001 = ***

Table 3A. Optimally fitted barrier items model including sociodemographic covariates and significant interaction terms

| Coefficients: | Odds | lower ci | upper ci | t value | Pr(>\|t\|) | Sig. |
| --- | --- | --- | --- | --- | --- | --- |
| (Intercept) | 1.614 | 1.349 | 1.931 | 5.234 | < .0001 | *** |
| Age | 0.980 | 0.977 | 0.983 | -13.355 | < .0001 | *** |
| BMI | 1.013 | 1.003 | 1.023 | 2.527 | 0.0117 | * |
| Less than high school | 0.799 | 0.668 | 0.956 | -2.45 | 0.0145 | * |
| Post-secondary edu | 1.244 | 1.098 | 1.410 | 3.423 | 0.0006 | *** |
| Income$0-29k | 0.766 | 0.648 | 0.905 | -3.139 | 0.0017 | ** |
| Income$30-59.9k | 0.798 | 0.683 | 0.932 | -2.851 | 0.0045 | ** |
| Income$100-150k | 0.938 | 0.809 | 1.088 | -0.841 | 0.4006 |  |
| Income$150k+ | 1.134 | 0.971 | 1.324 | 1.59 | 0.1121 |  |
| PerceivedHealthVeryPoor | 0.831 | 0.598 | 1.155 | -1.1 | 0.2717 |  |
| PerceivedHealthPoor | 1.102 | 0.911 | 1.334 | 0.998 | 0.3184 |  |
| PerceivedHealthVeryGood | 1.270 | 1.109 | 1.454 | 3.466 | 0.0006 | *** |
| PerceivedHealthExcellent | 1.345 | 1.150 | 1.574 | 3.711 | 0.0002 | *** |
| PreferPAbarrier | 0.535 | 0.448 | 0.639 | -6.926 | < .0001 | *** |
| ConfidencePAbarrier | 0.616 | 0.496 | 0.764 | -4.403 | < .0001 | *** |
| EnjoyPAbarrier | 0.717 | 0.580 | 0.885 | -3.09 | 0.0021 | ** |
| TimePAbarrier | 0.550 | 0.483 | 0.626 | -9.049 | < .0001 | *** |
| EnergyPAbarrier | 0.794 | 0.669 | 0.942 | -2.65 | 0.0082 | ** |
| SexFemale | 1.008 | 0.884 | 1.148 | 0.113 | 0.9102 |  |
| LocationRural | 1.266 | 1.070 | 1.500 | 2.733 | 0.0064 | ** |
| SeePAbarrier | 1.206 | 1.035 | 1.406 | 2.402 | 0.0165 | * |
| FacilitiesPAbarrier | 0.848 | 0.730 | 0.984 | -2.174 | 0.0299 | * |
| SupportPAbarrier | 0.993 | 0.842 | 1.171 | -0.086 | 0.9313 |  |
| Sex*Location | 0.695 | 0.562 | 0.860 | -3.343 | 0.0009 | *** |
| Location*SupportPAbarrier | 0.719 | 0.568 | 0.909 | -2.755 | 0.0060 | ** |
| Sex*SupportPAbarrier | 0.797 | 0.640 | 0.991 | -2.038 | 0.0418 | * |

NOTE: Intercept reflects odds reflecting the intersection of all regression anchors and as such the level of each variable serving as the regression anchor does not appear in the list of coefficients. See Table 1 for coding strategy. *p<*.05 = * ; *p*<.01 = ** ; *p*<.0001 = ***
